# Supplementary material for: Polyketide synthase gene clusters in the ericoid mycorrhizal fungus Oidiodendron maius: genome-wide prediction, expression profiling, and functional characterization of knockout mutants
Source: Front Plant Sci. 2026 Sep 16;17:1924430. doi: 10.3389/fpls.2026.1924430 (PMC13623903; doi:10.3389/fpls.2026.1924430)
Supplement: Supplementary file 1 [file SupplementaryFile1.zip › Supplementary Materials/Supplementary Folder 1.zip!/Oidma1_AssemblyScaffolds_Repeatmasked/knownclusterblast/cluster22/jgi.p_Oidma1_42740_mibig_hits.html]

| MIBiG Protein | Description | MIBiG Cluster | MiBiG Product | % ID | % Coverage | BLAST Score | E-value |
| --- | --- | --- | --- | --- | --- | --- | --- |
| ADN26241.1 | PLP-dependent\_aminotransferase | BGC0000951 | Pacidamycin\_biosynthetic\_gene\_cluster | 31 | 81.0810810811 | 170 | 6.7e-42 |
| ADY76679.1 | aminotransferase | BGC0000950 | Napsamycin\_biosynthetic\_gene\_cluster | 31 | 73.8045738046 | 165 | 1.7e-40 |
| EHN79454.1 | dTDP-4-keto-6-deoxy-glucose\_4-aminotransferase\_ScatV | BGC0000804 | Acarviostatin\_biosynthetic\_gene\_cluster | 29 | 87.525987526 | 140 | 4.4e-33 |
| ACH85568.1 | CetH | BGC0000283 | Cetoniacytone\_A\_biosynthetic\_gene\_cluster | 30 | 72.7650727651 | 136 | 8.2e-32 |
| BAO98809.1 | putative\_aminotransferase | BGC0001002 | Jawsamycin\_biosynthetic\_gene\_cluster | 29 | 73.1808731809 | 129 | 7.7e-30 |
| ADC96664.1 | aminotransferase | BGC0001076 | Liposidomycin\_biosynthetic\_gene\_cluster | 27 | 87.3180873181 | 128 | 2.2e-29 |
| ACQ63626.1 | aminotransferase | BGC0000875 | Caprazamycin\_biosynthetic\_gene\_cluster | 26 | 89.604989605 | 125 | 1.4e-28 |
| BAJ05891.1 | putative\_aminotransferase | BGC0000872 | A-90289\_biosynthetic\_gene\_cluster | 28 | 82.5363825364 | 124 | 2.5e-28 |
| ADD82957.1 | BatP | BGC0001099 | Kalimantacin\_/\_batumin\_biosynthetic\_gene\_cluster | 27 | 89.8128898129 | 103 | 7.7e-22 |
